# Discovery of ${{\bf{H}}}_{\mathbf{3}}^{\mathbf{+}}$ and infrared aurorae at Neptune with JWST

**DOI:** 10.1038/s41550-025-02507-9

**Published:** 2025-03-26

**Authors:** Henrik Melin, Luke Moore, Leigh N. Fletcher, Heidi B. Hammel, James O’Donoghue, Tom S. Stallard, Stephanie N. Milam, Michael Roman, Oliver R. T. King, Naomi Rowe-Gurney, Emma E. Thomas, Ruoyan Wang, Paola I. Tiranti, Jake Harkett, Katie L. Knowles

**Affiliations:** 1 Department of Maths, Physics, and Electrical Engineering, Northumbria University, Newcastle upon Tyne, UK; 2 Department of Astronomy, Boston University, Boston, MA USA; 3 Center for Space Physics, Boston University, Boston, MA USA; 4 School of Physics & Astronomy, University of Leicester, Leicester, UK; 5 Association of Universities for Research in Astronomy, Washington, DC USA; 6 Department of Meteorology, University of Reading, Reading, UK; 7 Department of Solar System Science, JAXA Institute of Space and Astronautical Science, Sagamihara, Japan; 8 NASA Goddard Space Flight Center, Greenbelt, MD USA

**Keywords:** Giant planets, Aurora

## Abstract

Emissions from the upper-atmospheric molecular ion ${{\rm{H}}}_{3}^{+}$ have been used to study the global-scale interactions of Jupiter, Saturn and Uranus with their surrounding space environments for over 30 years, revealing the processes shaping the aurorae. However, despite repeated attempts, and contrary to models that predict it should be present, this ion has proven elusive at Neptune. Here, using observations from the James Webb Space Telescope, we detect ${{\rm{H}}}_{3}^{+}$ at Neptune, as well as distinct infrared southern auroral emissions. The average upper-atmosphere temperature is a factor of two cooler than those derived 34 years ago by Voyager 2, showing that the energy balance of this region is regulated by physical processes acting on a timescale shorter than both Neptunian seasons (40 yr) and the solar cycle.

## Abstract (web summary)

JWST has detected ${{\rm{H}}}_{3}^{+}$ and auroral emissions at Neptune—the only giant planet in the Solar System for which they had proved to be elusive up to now. The observations reveal a factor-of-two cooling of Neptune’s upper atmosphere compared with Voyager 2 data, indicative of energy balance processes acting on a shorter timescale than solar forcing.

## Main

${{\rm{H}}}_{3}^{+}$ was detected outside the laboratory at Jupiter, Uranus and Saturn more than 30 years ago^1–3^. It is the dominant molecular ion in hydrogen atmospheres^4^ and the most prevalent molecular ion in interstellar space^5^. This ion, along with H^+^, constitutes the majority of the ionization in any giant-planet upper atmosphere, forming the ionosphere. This region is the medium through which energy and momentum is transferred between the magnetosphere and the underlying atmosphere, and observation of ${{\rm{H}}}_{3}^{+}$ is one of the only ways to reveal the chemistry and processes that govern these interactions^6–10^. ${{\rm{H}}}_{3}^{+}$ has remained undetected at Neptune, which is surprising, given that numerical models predict it to be readily observable^11,12^ at an altitude of >1,000 km, and there have been more than 30 years of unsuccessful attempts at detecting the ion^3,13–15^. ${{\rm{H}}}_{3}^{+}$ is expected to be produced from the ambient H_2_ population in the upper atmosphere, either via solar photoionization on the dayside, or via particle impact ionization about the magnetic poles, generally in the form of auroral electron precipitation. Due to their highly offset and complex magnetic fields^16^, aurorae at the ice giants are probably very different from those seen at both Jupiter and Saturn^17^. To understand the auroral process, the magnetic field, and how these couple to the lower atmosphere, all integral components of the Neptune system, detecting and mapping ${{\rm{H}}}_{3}^{+}$ is of utmost priority.

The spatial distribution of auroral emissions on a planet is a direct projection of the processes within the magnetic field that are responsible for generating them, shaped by factors such as magnetic field topology, sources of plasma, and planetary rotation rate^18^. At Earth, the particles responsible for the excitation in the polar atmosphere are primarily sourced from the solar wind, which enters the magnetosphere via reconnection on the magnetopause^19,20^. At Jupiter, the main auroral emission magnetically maps directly to a region well inside the magnetosphere, which may be associated with the breakdown of co-rotation of plasma sourced from the volcanic moon Io^21^, or by alternative mechanisms, such as wave–particle interactions above the atmosphere^22,23^. In this manner, the study of auroral morphology provides a remote diagnostic for the processes that occur inside the magnetosphere, and reveals the potential for internal plasma sources, such as geologically active moons.

Voyager 2 encountered Neptune in August 1989. The spacecraft did not carry a near-infrared spectrograph so was incapable of measuring ${{\rm{H}}}_{3}^{+}$, and only very tentative evidence for ultraviolet auroral emissions was present on the nightside^24^. This was supposedly consistent with the location of the northern auroral oval at longitude ~60° W, even though the scatter of the emissions across longitudes was substantial. Radio emissions were more revealing, showing smooth kilometric radiation (with time) above both magnetic poles (~60° N and ~35° S), whilst bursty emission was seen from above the southern pole only, where the higher field strength supports cyclotron maser instabilities^17^. These types of radio emission are indicative of plasma acceleration processes, required for the generation of the aurorae, suggesting the possibility of localized ${{\rm{H}}}_{3}^{+}$ auroral emissions. These are the only indicative evidence to date of the presence of auroral activity at Neptune.

Motivated by the severe lack of understanding of the ionospheric and auroral processes at Neptune, we used the James Webb Space Telescope (JWST) to observe Neptune in the near-infrared. JWST NIRSpec Integral Field Unit (IFU)^25^ observations of Neptune were obtained on 22 June 2023, covering two different central meridian longitudes (hereafter longitude 1 and longitude 2), separated by 172° longitude, capturing the two hemispheres of the planet. These observations include the spectral region between 2.87 and 5.27 μm, a wavelength region that contains discrete rovibrational ${{\rm{H}}}_{3}^{+}$ emission lines.

**Fig. 1:**
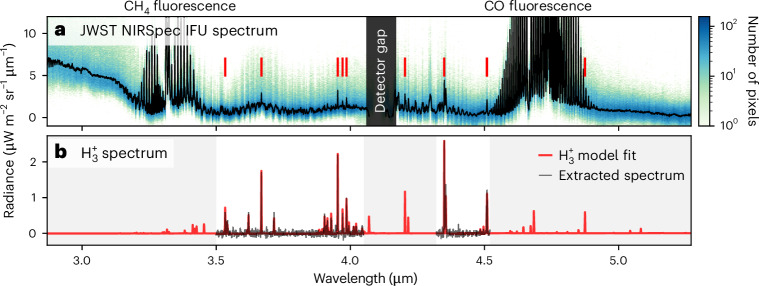
Detection of ${{\rm{H}}}_{3}^{+}$ at Neptune. **a**, The disk-median JWST NIRSpec spectrum from the two observed longitude sectors is shown in black, avoiding regions with bright 3 μm clouds, totalling 580 spaxels for both longitudes. The spectral pixel data density is shown as the green-to-blue background according to the colour bar. The red lines indicate the positions of bright ${{\rm{H}}}_{3}^{+}$ lines, clearly present in the observed spectrum. **b**, The background-subtracted spectrum (black), revealing clear discrete emission lines of ${{\rm{H}}}_{3}^{+}$. Only regions where the subtraction works well are shown, and regions outside this are shaded grey. The ${{\rm{H}}}_{3}^{+}$ spectrum fits to a temperature of 358 ± 8 K and an ion column density of (7.2 ± 1.4) × 10^14^ m^−2^.

**Fig. 2:**
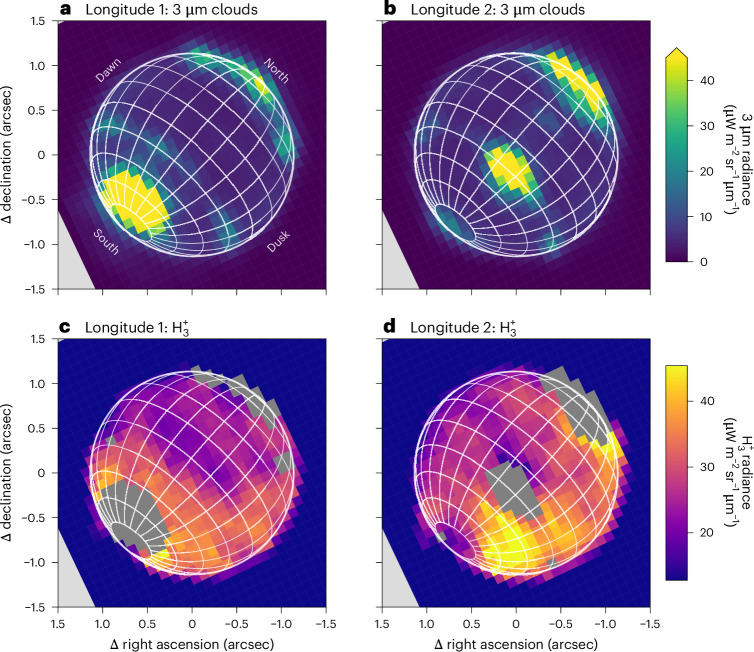
The JWST NIRSpec observations of Neptune. **a**,**b**, The spatial distribution of reflective clouds, seen clearly at 3 μm across the disk for longitude 1 (**a**) and longitude 2 (**b**). The latitude and longitude grids have spacings of 15°. **c**,**d**, The spatial distribution of the sum of the brightest ${{\rm{H}}}_{3}^{+}$ emission lines in Fig. 1. The grey areas show the regions of highly reflective clouds from which the ${{\rm{H}}}_{3}^{+}$ intensity cannot be extracted. **d** shows an enhancement in the southern hemisphere, appearing in the post-noon sector on the right. This is generated by enhanced ${{\rm{H}}}_{3}^{+}$ column density, probably indicative of localized auroral precipitation.

Figure 1a shows the disk-median near-infrared spectrum of Neptune (black) for the two longitudes, omitting regions of highly reflective clouds (Fig. 2a,b), with the data density shown as the blue background. There is variability across the disk, produced by the varying cloud reflectivity, which adds continuum to the spectral profile as the cloud brightness increases. There are also bright features of both methane and carbon monoxide fluorescence at ~3.3 μm and ~4.7 μm, respectively^13,26^. On top of the cloud reflectance spectrum sit discrete emission lines at wavelength locations matching exactly with those of ${{\rm{H}}}_{3}^{+}$ (red lines, with the model ${{\rm{H}}}_{3}^{+}$ spectrum shown in Fig. 1b). The spaxels containing very bright clouds, where the ${{\rm{H}}}_{3}^{+}$ component is swamped out, provide the overall shape of the continuum, which can be scaled to provide background subtraction for regions without bright clouds (see Methods for more details). The background-subtracted spectrum for wavelength regions where this method works well is shown in Fig. 1b, revealing a clear ${{\rm{H}}}_{3}^{+}$ spectrum. ${{\rm{H}}}_{3}^{+}$ has therefore been observed from Neptune. A spectral fit yields a globally averaged temperature of 358 ± 8 K of the upper atmosphere, and an ${{\rm{H}}}_{3}^{+}$ column integrated density of (7.2 ± 1.4) × 10^14^ m^−2^. The derived density is much smaller than seen at any other giant planet^9,27,28^, probably driven by the large heliospheric distance (29.9 AU), resulting in lower ionizing fluxes from the Sun, as predicted by modelling^12^. The ionosphere of Neptune is observed to be cooler than those of both Jupiter at ~700 K (ref. ^29^) and Saturn at ~500 K (ref. ^30^). The temperature is also lower than those observed at Uranus, which show unique behaviour, cooling slowly from ~750 K to ~450 K over three decades^9^, probably linked to the slowly changing seasons. Whilst Neptune receives ~3% of the solar flux of Jupiter, the retrieved temperature is still much higher than the ~130 K temperature that solar input alone can produce^31^. This highlights the giant-planet ‘energy crisis’, whereby the upper atmospheres of these planets have temperatures far in excess of that which can be explained by solar irradiance alone.

The retrieved globally averaged temperature is much cooler than found by the Voyager 2 ultraviolet occultation measurement, which found an exospheric temperature of 750 ± 150 K (ref. ^24^). On the basis of modelling^12^, the measured ${{\rm{H}}}_{3}^{+}$ temperature at Neptune is thought to be <10% lower than the equivalent temperature measured by Voyager 2, suggesting that the ionosphere was considerably hotter in 1989. Since the observed intensity of ${{\rm{H}}}_{3}^{+}$ is driven exponentially by temperature and only linearly by density, at 358 K the intensity of ${{\rm{H}}}_{3}^{+}$ is 0.8% of the intensity at 750 K for the same density, rendering the emissions extremely weak, and therefore very challenging to detect. The two latest attempts at detecting ${{\rm{H}}}_{3}^{+}$ from Neptune, using the 10 m Keck telescope^14^ and the 3 m NASA Infrared Telescope Facility^15^, both derived upper limits on the radiance that should have made it possible to detect the ion, given the strength of the emission observed with JWST. However, when observing the disk-integrated spectrum from the ground, it becomes dominated by the brightest cloud reflectivity, which markedly reduces the contrast of the ${{\rm{H}}}_{3}^{+}$ emissions. Here, the brightest clouds elevate the observed radiances at 3.9 μm to ~180 μW m^−2^ sr^−1^ μm^−1^, whereas the ${{\rm{H}}}_{3}^{+}$ Q(1, 0^−^) emission line peaks at ~2 μW m^−2^ sr^−1^ μm^−1^. This demonstrates the power of JWST, providing a combination of high spatial resolution and high sensitivity, not achievable at existing ground-based facilities.

The decrease in the temperature of Neptune’s upper atmosphere over only 34 yr is marked, and indicates changes on shorter timescales than the very long seasons, given that it takes the planet 165 yr to orbit the Sun. Similar changes have been observed in Uranus’s upper atmosphere^9,32–34^, and the ultimate cause of this remains unexplained. The changes in upper-atmosphere temperature are also unlikely to be related to the solar cycle. First, the energy crisis already demonstrates that solar flux has a very limited impact on the high observed temperatures^31^. Second, the mean composite solar H Lyman-α emission observed at Earth^35^ in the week surrounding the 1989 Voyager 2 encounter was 8.6 ± 0.4 mW m^−2^, whereas it was 9.6 ± 0.1 mW m^−2^ during the JWST observations, indicating comparable levels of solar activity. At Jupiter, auroral heating is thought to drive the variable global temperatures of the upper atmosphere^29^, and similar processes may be present at Neptune.

Whilst the signal-to-noise ratio of the ${{\rm{H}}}_{3}^{+}$ spectrum for individual spaxels is very low (~3), we can produce maps of the emission by extracting the peak radiance at the locations of the brightest ${{\rm{H}}}_{3}^{+}$ emission lines, and averaging each spaxel with the surrounding eight, shown in Fig. 2c,d. The uncertainty in the spectral radiance maps is ~5 μW m^−2^ sr^−1^ μm^−1^, on the basis of the s.d. of the off-disk noise. The morphology of these features is clearly not linked to the 3 μm cloud reflectivity features, showing a broad increase from dawn to dusk, that is, left to right, which may be consistent with the build-up of ${{\rm{H}}}_{3}^{+}$ across the dayside, a component likely linked to the solar extreme UV photoionization across the disk, as seen at Uranus^36^.

**Fig. 3:**
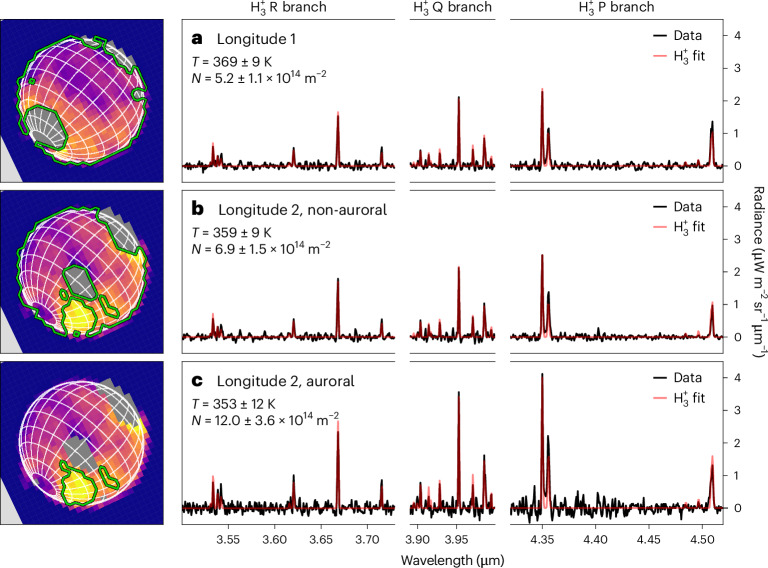
${{\rm{H}}}_{3}^{+}$ spectral fits at Neptune. **a**–**c**, Background-subtracted ${{\rm{H}}}_{3}^{+}$ spectral fits for three different locations on the planet away from bright 3 μm clouds: all of longitude 1 (**a**), all of longitude 2, apart from the subregion of enhanced ${{\rm{H}}}_{3}^{+}$ intensity (**b**), and the region of enhanced ${{\rm{H}}}_{3}^{+}$ intensity in longitude 2 (**c**). The images on the left of the figure show ${{\rm{H}}}_{3}^{+}$ intensity (from Fig. 2) and indicate the regions for which the medians were calculated for the extraction of the ${{\rm{H}}}_{3}^{+}$ spectrum. Here, we consider emissions from the P, Q and R branches of ${{\rm{H}}}_{3}^{+}$, referring to changes in the total angular momentum quantum number, Δ*J*, of −1, 0 and +1, respectively.

**Fig. 4:**
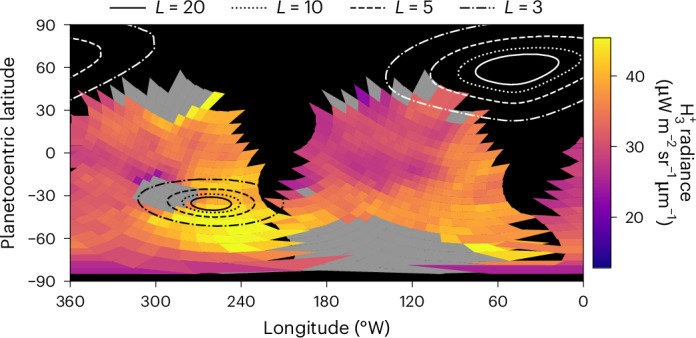
Projected ionospheric emissions at Neptune. The ${{\rm{H}}}_{3}^{+}$ observations of Fig. 2c,d projected to planetocentric latitude and west longitude. The contours represent magnetic *L* shells^39^, showing the magnetic mapping of the ionosphere (in *R*_*N*_). The positions of the auroral zones are a result of the offset and tilted magnetic field, which includes strong higher-order multipoles^41^. The localized enhancement of ${{\rm{H}}}_{3}^{+}$ located between 60° S and 30° S latitude and between 200° W and 280° W longitude coincides with the expected location in latitude of the southern aurora.

We detect an increase in the observed ${{\rm{H}}}_{3}^{+}$ radiance at a localized spot in longitude 2 located between 60° S and 30° S latitude and between 200° W and 280° W longitude, which is about twice as radiant as the surrounding regions. To determine what drives this increase we select the top quartile of radiances in the southern hemisphere, and create a median spectrum of this region, shown in Fig. 3c. The spectral retrieval reveals that this region has an ${{\rm{H}}}_{3}^{+}$ column density that is 1.7 times larger than rest of the observed disk ((12.0 ± 3.6) × 10^14^ m^−2^, see Fig. 3b), with the temperature being the same, within uncertainties, as that of the rest. A localized increase in column density is produced by localized enhancement in the H_2_ ionization, which is consistent with the presence of auroral precipitation, as observed at Jupiter, Saturn and Uranus^7,37,38^. In contrast, solar photoionization is expected to vary smoothly with solar zenith angle across the disk. The location of the bright ${{\rm{H}}}_{3}^{+}$ emission feature is consistent with the expected latitude location of the southern auroral oval^39^ (Fig. 4). The rotation rate of Neptune, 16.108 ± 0.006 h (ref. ^40^), has an uncertainty sufficiently large that the rotational phase is unknown at the current epoch, so the longitude of the magnetic pole can be arbitrarily shifted to match the observations. Surprisingly, there was no need to do this to achieve a good match, which may be a coincidence, or it could be indicative of overestimated uncertainties in the rotational period. The observed ${{\rm{H}}}_{3}^{+}$ enhancement is also in good agreement with the predicted longitude of the southern auroral emissions^39^.

The contours in Fig. 4 show the distances from the planet to which different locations magnetically map, known as *L* shells, as contours. For example, the *L* = 20 contours map to a distance of 20 *R*_*N*_. The southern ${{\rm{H}}}_{3}^{+}$ auroral emissions seen in Fig. 4 map to a range of *L* shells, ranging between <3 and 20 *R*_*N*_ southwards of the pole, and more confined between 3 and 5 *R*_*N*_ northwards of it, whereas the magnetopause sits between 26 and 34 *R*_*N*_ (ref. ^41^). Generally, this far out in the solar system, reconnection on the magnetopause is thought to be highly variable with seasonal geometry and magnetic field orientation^42^. However, Voyager 2 observed reconnection in situ on the dayside, providing the means to drive magnetospheric dynamics^43^, and providing a plasma source for the field-aligned currents driving the aurorae. Another potential source of plasma is the geologically active moon Triton, orbiting at 14.4 *R*_*N*_, which itself has a substantial ionosphere, providing a source of plasma inside the magnetosphere. The estimated neutral outflow rate is ~10^25^ atoms s^−1^ (ref. ^44^), which could undergo solar photoionization and charge exchange, analogous to processes seen for Io at Jupiter^45^ and Enceladus at Saturn^46^. The extent to which internal plasma loading is important at Neptune, versus plasma sourced from the solar wind, remains an open question^12^.

**Fig. 5:**
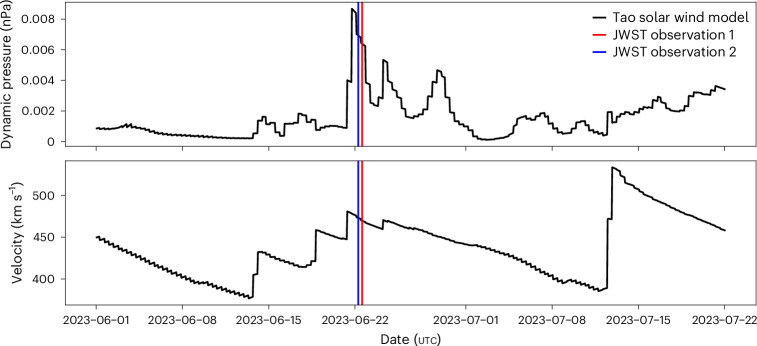
Solar-wind properties. The propagated solar-wind properties at Neptune during the JWST observations^47^. The vertical lines indicate the mid-point of the JWST NIRSpec observations detailed here.

The solar-wind properties at Neptune during the JWST observations can be estimated by using a propagation model^47^. Whilst the uncertainties of the arrival time are large (weeks), the observations coincide with an increase in the solar-wind dynamic pressure (Fig. 5), the sixth strongest predicted during 2023. At Earth, these compressions can drive strong magnetospheric convection and hence auroral emission towards lower altitudes^48^, and similar processes may occur at Neptune, driving emissions towards lower *L* shells.

The discovery of ${{\rm{H}}}_{3}^{+}$ and spatially resolved auroral emission from Neptune’s southern pole opens up a new chapter in understanding the outermost planet of the solar system, and how the ice giants couple with their surrounding space environments. The fact that the upper atmosphere of both ice giants can change markedly in temperature over timescales that are relatively short compared with their orbital periods has implications for the physical processes that occur within them. For example, the change in temperature from 750 K to 350 K implies that the atmospheric scale height has been reduced by a factor of over two, radically altering the vertical extent of the upper atmosphere. This in turn has consequences for the atmospheric drag and evolution of the inner rings, and can strongly modify the inflow from them^49^. Finally, since the most commonly detected type of extrasolar planet is Neptune sized^50^, and as Neptune lacks the extreme seasons of Uranus, these observations provide a new diagnostic to probe atmosphere–magnetosphere interactions on the most common-sized worlds in our galaxy.

## Methods

### Observations

JWST NIRSpec^25^ observations of Neptune were obtained on 2023-06-22 as part of the Solar System Guaranteed Time Observations awarded to H. Hammel (1249, principal investigator L. N. Fletcher) using the G395H/F290LP grating/filter setting, producing a spectrum between 2.87 and 5.27 μm at a resolving power of *R* ≈ 2,700. This wavelength region contains the brightest ${{\rm{H}}}_{3}^{+}$ emission lines (Fig. 1). Two individual observations were obtained, separated by 7.7 h (172° longitude), capturing almost complete global coverage. Neptune subtended 2.29″ in the sky, fitting comfortably within the 3″ × 3″ IFU. Each of the 30 × 30 IFU spaxels measures 0.1″ × 0.1″, producing a spatial resolution on the centre of the disk of about 2,150 km. The sub-JWST latitude was 20.2° S, the subsolar latitude was 20.8° S and the central meridian longitude (IAU) was 131.0° W and 303.2° W for longitude 1 and longitude 2, respectively. Each longitude was observed using four dithers, each using eight exposures with ten groups, with a total effective exposure time of ~57 min per longitude. The data were calibrated using the Calibration Reference Data System context jwst_1097.pmap and the jwst calibration pipeline v.1.11.0 with the level 3 coord_system option set to ifualign, with the dithers combined.

### Background subtraction and ${{\rm{H}}}_{3}^{+}$ fitting

Figure 2a,b shows bright 3 μm clouds on the disk of Neptune at both longitudes that are over 50 times brighter than the ${{\rm{H}}}_{3}^{+}$ emissions we are wanting to extract, and therefore extracting ionospheric emissions at these clouds is not possible. However, the bright clouds provide a high signal-to-noise spectrum of the cloud and aerosol reflectance contribution, containing only a minute ${{\rm{H}}}_{3}^{+}$ component (~2%), which can be used for background subtraction in the regions without bright clouds. We use the brightest 1% of cloud radiances to generate this background spectrum, which is then scaled to fit the background spectrum, on top of which ${{\rm{H}}}_{3}^{+}$ emission lines sit. Once the ${{\rm{H}}}_{3}^{+}$ spectrum has been isolated, it can be fitted using the open-source fitting procedure h3ppy.

## Data Availability

JWST data used in this study were obtained from the Mikulski Archive for Space Telescopes at the Space Telescope Science Institute (https://archive.stsci.edu/), which is operated by the Association of Universities for Research in Astronomy, Inc., under NASA contract NAS 5-03127 for the JWST. JWST NIRSpec Guaranteed Time Observation programme 1249 observations of Neptune are available at 10.17909/tn0h-ww73.

## Code availability

The open-source Python ${{\rm{H}}}_{3}^{+}$ fitting code h3ppy is available at https://github.com/henrikmelin/h3ppy/. The code (and data) to perform the analysis and generate the figures of this study is available at https://github.com/henrikmelin/neptune-h3p/.
